# Lack of Adrenomedullin Results in Microbiota Changes and Aggravates Azoxymethane and Dextran Sulfate Sodium-Induced Colitis in Mice

**DOI:** 10.3389/fphys.2016.00595

**Published:** 2016-11-30

**Authors:** Sonia Martínez-Herrero, Ignacio M. Larrayoz, Judit Narro-Íñiguez, María J. Villanueva-Millán, Emma Recio-Fernández, Patricia Pérez-Matute, José A. Oteo, Alfredo Martínez

**Affiliations:** ^1^Oncology Area, Center for Biomedical Research of La RiojaLogroño, Spain; ^2^Infectious Diseases Department, Center for Biomedical Research of La RiojaLogroño, Spain

**Keywords:** adrenomedullin, intestinal microbiota, inflammatory bowel disease, colitis, colorectal cancer

## Abstract

The link between intestinal inflammation, microbiota, and colorectal cancer is intriguing and the potential underlying mechanisms remain unknown. Here we evaluate the influence of adrenomedullin (AM) in microbiota composition and its impact on colitis with an inducible knockout (KO) mouse model for AM. Microbiota composition was analyzed in KO and wild type (WT) mice by massive sequencing. Colitis was induced in mice by administration of azoxymethane (AOM) followed by dextran sulfate sodium (DSS) in the drinking water. Colitis was evaluated using a clinical symptoms index, histopathological analyses, and qRT-PCR. Abrogation of the *adm* gene in the whole body was confirmed by PCR and qRT-PCR. KO mice exhibit significant changes in colonic microbiota: higher proportion of δ*-Proteobacteria* class; of *Coriobacteriales* order; and of other families and genera was observed in KO feces. Meanwhile these mice had a lower proportion of beneficial bacteria, such as *Lactobacillus gasseri* and *Bifidobacterium choerinum*. TLR4 gene expression was higher (*p* < 0.05) in KO animals. AM deficient mice treated with DSS exhibited a significantly worse colitis with profound weight loss, severe diarrhea, rectal bleeding, colonic inflammation, edema, infiltration, crypt destruction, and higher levels of pro-inflammatory cytokines. No changes were observed in the expression levels of adhesion molecules. In conclusion, we have shown that lack of AM leads to changes in gut microbiota population and in a worsening of colitis conditions, suggesting that endogenous AM is a protective mediator in this pathology.

## Introduction

Gastrointestinal diseases are emerging as an important global health problem (Ponder and Long, 2013). Many of them are idiopathic, chronic, relapsing disorders of the gastrointestinal tract, such as inflammatory bowel diseases (IBD), which encompass ulcerative colitis and Crohn's disease. IBD is also widely accepted as one of the main risk factors leading to colorectal cancer (CRC) (Kim and Chang, 2014), mediated by chronic intestinal inflammation (Yashiro, 2014).

Adrenomedullin (AM) and proadrenomedullin N-terminal 20 peptide (PAMP) are two biologically active peptides produced by the same gene, *adm*, with ubiquitous distribution and many physiological functions (López and Martinez, 2002). The AM receptor consists on a 7-transmembrane domain protein called calcitonin receptor-like receptor (CLR) in combination with a single transmembrane domain protein known as receptor activity modifying protein (RAMP) (Poyner et al., 2002). Specific binding and production sites for AM are located in many tissues and cell types (López and Martinez, 2002), including the gastrointestinal tract (Martínez-Herrero and Martínez, 2016), being especially abundant in the neuroendocrine cells of the gastrointestinal mucosa, suggesting that AM and PAMP may act as gut hormones regulating many physiological and pathological conditions. Furthermore, AM and PAMP are antimicrobial peptides which are found in mostly all epithelial surfaces and body secretions (Martínez et al., 1997) and it has been demonstrated that Gram-positive and Gram-negative bacteria isolated from the skin, oral cavity, and respiratory and gastrointestinal tract are sensitive to AM (Allaker et al., 1999).

AM has a protective role in gastrointestinal diseases, including IBD, and its administration in rodents (Ashizuka et al., 2009) and humans (Ashizuka et al., 2013, 2016) ameliorates the severity of these gut pathologies, emerging as a new promising therapeutic alternative. However, these results should be considered with care since IBD patients have an elevated risk of developing colitis-associated CRC, and the involvement of AM in tumor progression has become evident in the last years (Larráyoz et al., 2014). In the particular case of CRC, several studies postulate that blocking AM might prevent tumor growth (Nougueréde et al., 2013).

In susceptible hosts an abnormal communication between gut microbial communities and the mucosal immune system has been identified as the core defect that leads to chronic intestinal inflammation (Leone et al., 2013). In normal gut, intestinal epithelial cells maintain a beneficial link with the microorganisms in the intestinal flora through toll-like receptors (TLRs), which mediate signaling to maintain epithelial cell integrity and tight junctions (Yesudhas et al., 2014). Stimulation from commensal bacteria is finite and should not trigger an excessive inflammatory response. However, any alteration in the population of luminal bacteria may influence TLR signaling, paving the way to a dysregulated inflammatory response, thus being a common denominator of IBD and CRC (Ha et al., 2014). Altered expression patterns of TLRs lead to tumor development, and in this context the contribution of TLR4 is considerably higher than those of other TLRs (Yesudhas et al., 2014).

To carry out formal studies on the correlation between AM and microbiota composition and the impact of these changes in colitis and colitis-associated CRC *in vivo*, a knockout (KO) model for either AM or its receptors is needed. Previous results have shown that early abrogation of the gene coding for AM or some of its receptor components results in 100% embryo lethality due to serious vascular abnormalities (Shindo et al., 2001). To circumvent this problem, we developed a conditional knockout for AM using *Cre/loxP* technology and have shown that it works well when targeting specific cell types such as neurons (Fernández et al., 2008), endothelial cells (Koyama et al., 2013), or pulmonary club cells (García-Sanmartín et al., 2016). Recently we have generated an inducible model in which the AM gene can be eliminated in adult mice (Martínez-Herrero et al., 2016). These animals survive whole body abrogation of the gene and constitute a perfect model to study the physiological implications of AM, including the impact of the total lack of AM and PAMP on gut microbiota, colitis, and CRC initiation.

## Materials and methods

### Generation and characterization of inducible knockout mice

All procedures involving animals were carried out in accordance with the European Communities Council Directive (2010/63/UE) and Spanish legislation (RD53/2013) on animal experiments and with approval from the ethical committee on animal welfare of our institution (Órgano Encargado del Bienestar Animal del Centro de Investigación Biomédica de La Rioja, OEBA-CIBIR).

An inducible KO mouse model for AM was developed and characterized in our laboratory (Martínez-Herrero et al., 2016). For experiments, the following 2 genotypes were selected: normal wild type (WT) controls (homozygous for the *adm* wild type allele, tetO-Cre, and rtTA) and KO animals (homozygous for the “floxed” *adm* allele, tetO-Cre, and rtTA). Male and female, 9 week-old, mice of both genotypes were exposed to 2 mg/ml doxycycline in the drinking water, supplemented with 5% sucrose, for 15 days. After this period, mice were allowed to rest for at least 4 weeks before performing any experiments. At this point, a group of animals (*n* = 5 per sex and genotype) were sacrificed and several organs collected and frozen in liquid N_2_. DNA was extracted and subjected to PCR with primers located outside the *loxP* sequences: P1 (sense): AAGGGAAGTCCTGCTCCAGT, and P2 (antisense): GCCTTAGCTCAGGTCCAGTG. The expected amplicon size is 2500 bp for the WT allele and 600 bp after the *adm* gene has been eliminated.

### Feces collection and DNA extraction

Twenty eight mice (6 months-old) were divided in: WT males (WTM), WT females (WTF), KO males (KOM), and KO females (KOF). All of them were treated with doxycycline, were fed the same diet, housed in the same room under specific-pathogen-free (SPF) conditions, and maintained by the same personnel in order to normalize their microbiota. Fresh fecal contents were collected from each animal and weighed. DNA was subsequently extracted from fecal microbiota using the DNeasy Blood & Tissue Kit (Qiagen, Venlo, Netherlands). DNA purity and concentration were determined by a Nanodrop spectrophotometer (ND-1000; Thermo Fisher Scientific, Waltham, MA).

### Bacterial 16S rDNA massive sequencing and sequence postprocessing

Samples were amplified for the 16S rDNA hypervariable sequence V4 using previously described primers (515F-806R) (Caporaso et al., 2012) in a MiSeq Instrument (2 × 150 bp reads) (Illumina, INC, San Diego, CA). Nucleotide filiations were assigned using the Ribosomal Database Project (RDP) (Cole et al., 2014). Two different taxonomic assignment approaches were used: BBH (*Best Blast Hit*: each read was assigned to the taxon corresponding to the Best Blast Hit over a threshold of similarity) and LCA (*Lowest Common Ancestor*: adopted by advanced tools of metagenomics analysis such as the last version of MEGAN Huson and Weber, 2013). The direct assignments (calculated as counting reads specifically assigned to a node, not including the reads assigned to the descendant nodes in the taxonomy tree) and the cumulative assignment frequencies (calculated by including the direct frequencies and also the frequencies of the descendant nodes) for each taxonomy node (with some read assigned) were analyzed. A β-diversity analysis was carried out in order to deeply analyse the distinctness among communities (Pasari et al., 2013). The METAGENassist web server was used for comparative metagenomics (Arndt et al., 2012).

### Colitis-associated cancer induction

Fifty seven 16-week old mice were used in this study. All of them had been previously treated with doxycycline. The groups were formed as follow: control WTM (*n* = 5), treated WTM (*n* = 10), control KOM (*n* = 5), treated KOM (*n* = 9), control WTF (*n* = 4), treated WTF (*n* = 10), control KOF (*n* = 4), and treated KOF (*n* = 10). The protocol was performed as previously described (Thaker et al., 2012). Briefly, treated animals received a single intraperitoneal injection (10 mg/Kg) of the carcinogen azoxymethane (AOM) (Sigma-Aldrich, Madrid, Spain). One week later, animals were given 2.5% dextran sulfate sodium (DSS) (Sigma-Aldrich) in the drinking water for 1 week followed by 2 weeks of tap water. The DSS treatment should be repeated for 2 additional cycles and tumorigenesis should be examined 2 weeks after the last cycle. However, KO animals experienced an unexpected overreaction to the first DSS cycle and the procedure had to be terminated at this point according to the animal welfare guidelines because humane endpoints were reached. Untreated control mice received a saline injection instead of AOM and drank tap water only.

### Clinical assessment of colitis

Mice were weighed and observed daily. Assessments of rectal bleeding, diarrhea, prolapse, inactivity, and percent weight loss relative to baseline were scored according to the system described by Gommeaux et al. (2007) and used as a surrogate measure of colitis severity.

### Mouse sacrifice, macroscopic analysis, and tissue harvesting

All mice were sacrificed by an overdose of anesthesia 14 days (males) and 15 days (females) after AOM injection according to humane endpoint. Entire colons were dissected, rinsed with ice-cold phosphate buffer solution (PBS) to remove fecal residues, and weighed. Photographs of colon samples were taken using an *EOS 50D* camera (Canon, Tokyo, Japan). Colon fragments were snap-frozen in liquid N_2_ and stored at −80°C for further analysis. Central portions of colonic tissue were fixed in 10% buffered formalin, processed for paraffin embedding, and sections were stained with hematoxylin and eosin (H&E) or with immunohistochemical techniques.

### Histopathological studies of the colon

Three H&E-stained sections from each colon sample were used for histological evaluation of colonic damage. The slides were coded to prevent observer bias during evaluation. All sections were examined in an Eclipse 50i microscope (Nikon, Amsterdam, Netherlands). FIJI software was used for characterization of histopathological changes. The height of the mucosa and the submucosa were used as a surrogate measure of inflammation. In addition, tissues were scored using the histopathological colitis scoring method, as described (Hayashi et al., 2011).

### Immunohistochemistry

Tissue sections (3 μm-thick) were dewaxed, exposed for 15 min to 3% H_2_O_2_ in methanol to block intrinsic peroxidase, rehydrated, and subjected to antigen retrieval (10 mM Sodium Citrate, 0.5% Tween 20, pH 6.0, 20 min at 95°C). Non-specific binding was blocked by exposure to 10% normal donkey serum (Jackson Immunoresearch Laboratories, West Grove, PA) for 30 min, and then to 10% Fab fragment donkey anti-mouse IgG (Jackson) for 1 h to block potential mouse IgGs in the section. Then tissue sections were incubated with primary mouse monoclonal antibody anti-TLR4 (sc-293072, Santa Cruz, Dallas, TX) overnight at 4°C. The following day, the presence of the primary antibody was revealed by a biotinylated donkey anti-mouse antibody (Jackson), the Vectastain Elite ABC Peroxidase Kit (Vector Laboratories, Burlingame, CA), and diaminobenzidine (Sigma). Sections were lightly counterstained with hematoxylin. Adjacent sections were incubated in the absence of primary antibody as a control.

### RNA isolation and quantitative real-time PCR

RNA isolation, cDNA synthesis, and qRT-PCR were performed as described (Larrayoz et al., 2012). Briefly, total RNA was isolated from distal colon fragments using Qiagen RNAseasy Mini Kit with DNAse digestion step performed (Qiagen, Hilden, Germany) according to manufacturer's instructions. Total RNA (1 μg) of each sample was reverse transcribed using the SuperScript® III Reverse Transcriptase Kit (Thermo Fisher Scientific). The synthesized cDNA was amplified by qRT-PCR with a 7300 real-time PCR System (Applied Biosystems, Foster City, CA, USA) and gene expression was calculated using absolute quantification by interpolation into a standard curve using RQ software (Applied Biosystems), as described (Schmittgen and Livak, 2008). All values were divided by the expression of the house keeping gene, 18S, to avoid potential loading errors. Target genes (AM, TNF-α, IL-1β, IL-6, IL-10, IL-17, IL-22, ZO-1, occluding, JAM-A, β-catenin, E-cadherin, desmoglein-2, connexin 26, β-actin, and TLR4) and primers are described in Table 1 of Supplementary Material.

### Protein extraction and western blotting

Small fragments of colon were homogenized (1:3, w/v) in lysis buffer (20 mM HEPES, 0.2 M sucrose, 5 mM DTT, 1 mM ethylenediaminetetraacetic acid (EDTA), 10 μg/ml soybean trypsin, 10 μg/ml leupeptin, 2 μg/ml pepstatin, 0.1 mM PMSF, pH 7.4) at 4°C. Homogenates were centrifuged for 30 min at 15,000 × g and the supernatants collected. Protein concentration was determined by the BCA kit (Pierce, Rockford, IL), with bovine serum albumin as standard, using a NanoDrop spectrophotometer (ND100). Then, 25 μg of each sample were mixed with 4x sample buffer (Invitrogen) and heated for 10 min at 70°C. Samples were run on 10% SDS–polyacrylamide gels. Seeblue plus 2 Prestained Standards (Invitrogen) were used as molecular weight markers. For Western blot analysis, proteins were transferred onto 0.2-μm polyvinylidene difluoride (PVDF) membranes (iBlot system, Invitrogen). For protein identification, membranes were incubated overnight at 4°C with primary monoclonal antibody anti-TLR4 (sc-293072, Santa Cruz) at a dilution 1:200. To standardize the results, a monoclonal IgG anti-β-actin antibody (Sigma) was used at a dilution 1:10,000 in the same membranes. To visualize immunoreactivity, membranes were incubated with anti-mouse peroxidase- labeled IgGs, developed with a chemoluminiscence kit (GE Biosciences, Miami, FL), and exposed to X-ray films (GE Biosciences). Developed films were scanned with a computer-assisted densitometer (GS-800, Bio-Rad) and optical density quantified by NIH ImageJ software.

### Statistical analysis

All data sets were analyzed for normalcy and homoscedasticity. Normal data were analyzed by Unpaired Student's *t*-test or by 1-way ANOVA followed by Tukey's Multiple Comparison Test. Data that did not follow a normal distribution were compared by Kruskal-Wallis test followed by Dunn's *Post-hoc* Test. All these studies were performed with GraphPad Prism version 5.02 (GraphPad Software, Inc. La Jolla, CA). A *p* < 0.05 was considered statistically significant. Metagenomic results were analyzed by Wilcoxon test using SPSS version 17.0 (SPSS® Inc. Chicago, IL).

## Results

### Characterization of the inducible knockout

**Figure 1:**
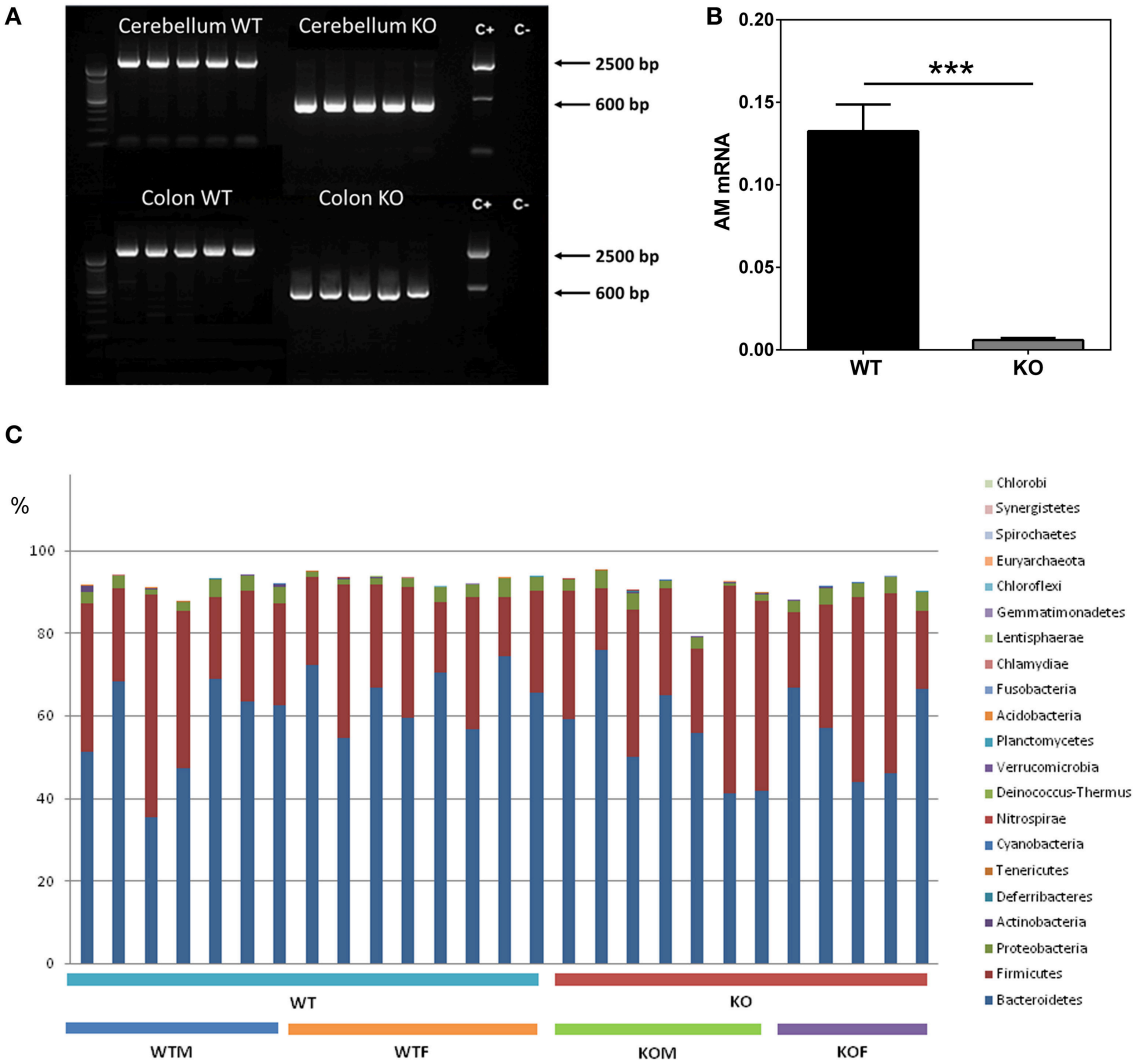
**Characterization of the knockout phenotype and analysis of gut bacterial communities by 16S rDNA sequencing**. Cerebellum and colon **(A)** fresh tissue was collected from 5 WT and 5 KO mice and subjected to PCR with primers which distinguish the unmodified *adm* gene (~2500 bp) from the recombined allele (~600 bp). Doxycycline administration effectively suppresses AM expression in colonic tissues **(B)**. After DSS treatment, RNA was extracted from colonic biopsy specimens and analyzed for AM levels by qRT PCR. Panel **(C)** shows the percentages which each community contributed by the indicated Phyla. Data are shown as mean ± SEM. Kruskal-Wallis test; ^***^P < 0.001; 29 WT and 28 KO mice have been included. WT, Wild type mice; KO, Knockout mice; WTM, male wild type mice; WTF, female wild type mice; KOM, male knockout mice; KOF, female knockout mice.

Mice with the proper genotypes were obtained and subjected to doxycycline treatment for 2 weeks. Confirmation of *adm* deletion was performed by PCR. In all the WT animals a band of around 2500 bp was obtained, indicating the presence of the gene despite the treatment with doxycycline. In contrast, all tissues obtained from the KO mice presented a single band of approximately 600 bp, showing a complete deletion of *adm* (Figure 1A).

Analysis of AM mRNA levels confirmed *adm* abrogation and demonstrated a very significant reduction of this molecule in the colon of KO mice (*n* = 28) when compared with WT animals (*n* = 29) (*p* < 0.001) (Figure 1B).

### Metagenomic analysis of gut bacterial populations from WT and KO mice

Using our doxycycline-inducible KO model we have studied the effects of eliminating the *adm* gene on gut microbiota composition under physiological conditions.

Figure 1C shows the relative abundance of the major bacterial phyla present in the gut. As expected, around 90% of the bacteria detected (89.4 and 87.3% for WT and KO animals respectively) belong to the *Bacteroidetes* and *Firmicutes* phyla (Table 1). At phylum level, no significant differences were observed in the most abundant phyla in the gut between WT and KO mice, although a slight decrease in the abundance of the *Bacteroidetes* was observed in KO animals (*p* = 0.07) (Table 1). However, significant changes were observed in other phyla, less represented in the gut (Table 1). Some of these differences were sex dependent. Thus, a significant reduction in the abundance of *Deferribacteres* was observed in WTF when compared with WTM, and an opposite tendency was obtained when comparing the KO groups. The abundance of *Tenericutes* was significantly higher in WTF in comparison with WTM, whereas the abundance of this phylum was slightly decreased in KOF when compared to KOM (Table 1).

**Table 1:** **Relative abundance of the most representative phyla in the gut of wildtype (WT) male and female mice and of knockout (KO) male and female mice**.

	**Taxonomic group (relative abundance [%])**	**WT**		**KO**		**Overall *p* value^†^**
		**Male**	**Female**	**Male**	**Female**	
Phyla	Firmicutes	31.68 ± 4.513	25.45 ± 2.770	31.99 ± 4.867	31.05 ± 5.736	0.722
	Bacteroidetes	56.61 ± 4.717	64.92 ± 2.621	55.48 ± 4.743	55.99 ± 4.850	0.282
	Proteobacteria	3.127 ± 0.403	2.619 ± 0.405	2.652 ± 0.476	3.721 ± 0.297	0.349
	α-Proteobacteria^@^	0.131 ± 0.050	0.023 ± 0.004	0.038 ± 0.012	0.033 ± 0.015	0.147
	β-Proteobacteria^@^	0.167 ± 0.012	0.113 ± 0.014	0.110 ± 0.019	0.081 ± 0.041	0.086
	γ-Proteobacteria^@^	0.018 ± 0.004	0.040 ± 0.013	0.025 ± 0.003	0.271 ± 0.116	0.114
	δ-Proteobacteria^@^	0.419 ± 0.141	0.478 ± 0.056	0.736 ± 0.157	1.532 ± 0.129^#^	**0.013**
	Actinobacteria	0.231 ± 0.116	0.220 ± 0.038	0.198 ± 0.041	0.322 ± 0.054	0.227
	Deferribacteres	0.107 ± 0.026	0.022 ± 0.009^*^	0.029 ± 0.014	0.054 ± 0.033	**0.026**
	Tenericutes	0.002 ± 0.001	0.072 ± 0.020^*^	0.058 ± 0.026	0.011 ± 0.008	**0.012**
Ratios	Bacteroidetes/Firmicutes	2.104 ± 0.390	2.880 ± 0.446	1.712 ± 0.329	2.223 ± 0.579	0.327
	Proteobacteria/Firmicutes	0.120 ± 0.026	0.123 ± 0.033	0.079 ± 0.019	0.139 ± 0.031	0.513
	Actinobacteria/Firmicutes	0.014 ± 0.007	0.007 ± 0.001	0.005 ± 0.001	0.009 ± 0.002	0.508

*Data are presented as mean values ± SEM*.

* p < 0.05 vs. WTM;

# *p < 0.05 vs. WTF*.

@ *Bacterial classes belonging to Proteobacteria phylum*.

† *Statistically significant differences are indicated by bold type*.

At the class level, AM deletion resulted in a significant increase in the abundance of the δ*-Proteobacteria*, especially in KOF mice (*p* < 0.05 vs. WTF mice) (Table 1). A slight decrease was also observed in the abundance of β*-proteobacteria* class in KO animals (*p* = 0.086) (Table 1).

At the order level, KO mice showed a significant increase in the abundance of *Coriobacteriales*, whereas a significant reduction in the abundance of *Erysipelotrichales* and *Caulobacterales* was observed (Table 2). The increase observed in the abundance of *Coriobacteriales* was similar in both males and females (Table 2, Figure 1A of Supplementary Material) whereas the decrease observed in *Erysipelotrichales* was restricted to males (Figure 1B of Supplementary Material). In contrast, the decrease in the abundance of *Caulobacterales* was only observed in KOF mice but not in KOM (Figure 1C of Supplementary Material). Although no statistical differences were observed in the abundance of *Anaeroplasmatales* when comparing WT vs. KO mice, it is worth mentioning the significant increase observed in the abundance of this bacterial order in KOM mice and WTF in comparison with their corresponding sex controls (Figure 1D of Supplementary Material).

**Table 2:** **Abundance of lower taxonomic levels (order, family, and genus) which were significantly increased or decreased in feces from knockout (KO) mice compared to wild type (WT) animals**.

**Phylum**	**Taxonomic group**	**Category**	**Variation**	**p value**	**Effects by sex**
*Actinobacteria*	*Coriobacteriales*	Order	Increased	0.005	both
*Bacteroidetes*	*Bacteroidaceae*	Family	Increased	0.028	–
*Bacteroidetes*	*Rikenellaceae*	Family	Increased	0.0002	both
*Proteobacteria*	*Pseudomonadaceae*	Family	Increased	0.048	–
*Firmicutes*	*Clostridium*	Genus	Increased	0.025	–
*Firmicutes*	*Lactococcus*	Genus	Increased	0.047	male
*Bacteroidetes*	*Odoribacter*	Genus	Increased	0.032	–
*Bacteroidetes*	*Alistipes*	Genus	Increased	0.0001	female
*Firmicutes*	*Erysipelotrichales*	Order	Decreased	0.020	male
*Proteobacteria*	*Caulobacterales*	Order	Decreased	0.003	female
*Actinobacteria*	*Bifidobacterium*	Genus	Decreased	0.004	male
*Actinobacteria*	*Olsenella*	Genus	Decreased	0.05	female
*Proteobacteria*	*Brevundimonas*	Genus	Decreased	0.013	female

*Values obtained by BBH alignment assay*.

At the family level, KO mice showed a significant increase in *Bacteroidaceae, Pseudomonadaceae*, and *Rikenellaceae* families with no significant differences according to sex (Table 2).

**Figure 2:**
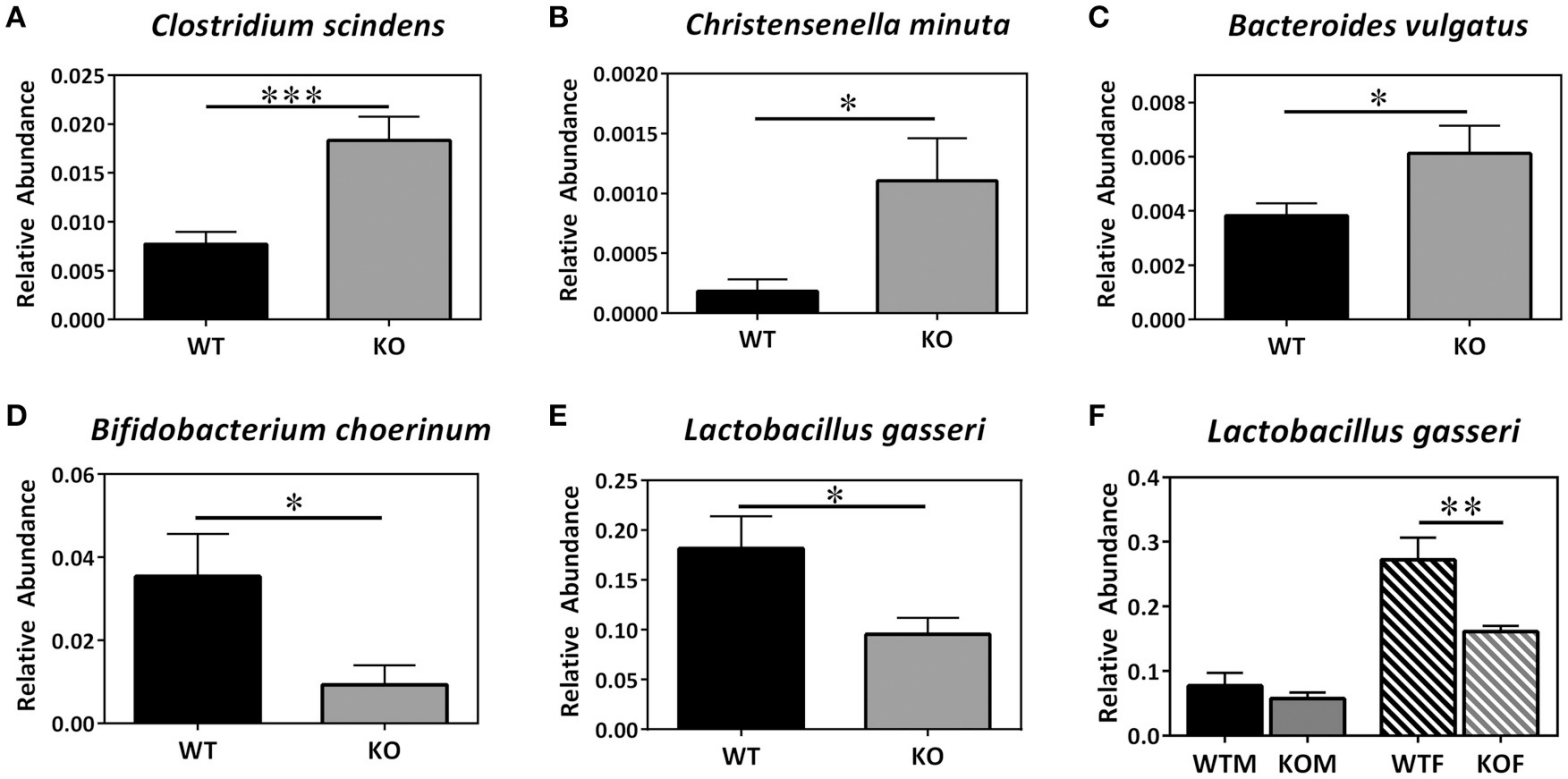
**Metagenomic analysis of bacterial species in the colonic microbiota of experimental animals**. Several bacterial species **(A–E)** are shown where statistically significant differences in abundance were found between genotypes. WT, Wild type mice; KO, Knockout mice. In the case of *L. gasseri* an additional panel **(F)** was added to point out the sex influence. Data are shown as mean ± SEM. Kruskal-Wallis test; ^*^P < 0.05; ^**^P < 0.001; ^***^P < 0.001. 20-week old WT (*n* = 8) and KO (*n* = 8) mice have been included.

Concerning lower taxonomic levels (genera), KO mice exhibited a significant increase in the abundance of *Clostridium, Lactococcus, Odoribacter*, and *Alistipes* and a significant decrease in the presence of bacteria belonging to the genera *Bifidobacterium, Olsenella*, and *Brevundimonas* (Table 2). The increase observed in *Lactococcus* was more evident in males than in females; however, the increase in *Alistipes* was only observed in females. In addition, the decrease observed in *Bifidobacterium* was only evident in males and, on the contrary, the decrease observed in *Olsenella* and *Brevundimonas* were only evident in females. Although, no differences were observed in the abundance of *Acinetobacter* genus when comparing the KOs vs. their corresponding WT, a significant increase in the abundance of this genus was observed in KOM in comparison with their respective WT group. Finally, the abundance of several bacterial species was also detected using the BBH alignment assignment. Thus, a significant higher presence of *Clostridium scindens, Christensenella minuta*, and *Bacteroides vulgatus* was observed in KO mice (Figures 2A–C). A tendency to higher abundance of *Lachnospiraceae bacterium* and *Faecalibacterium prausniztii* in KO mice were also detected although it did not reach statistical significance (Figure 2 of Supplementary Material). On the other hand, decreased abundance of *Bifidobacterium choerinum* and *Lactobacillus gasseri* (Figures 2D–F) was detected in feces from KO mice in comparison with WT animals.

### KO mice express higher levels of TLR4 in the colon

**Figure 3:**
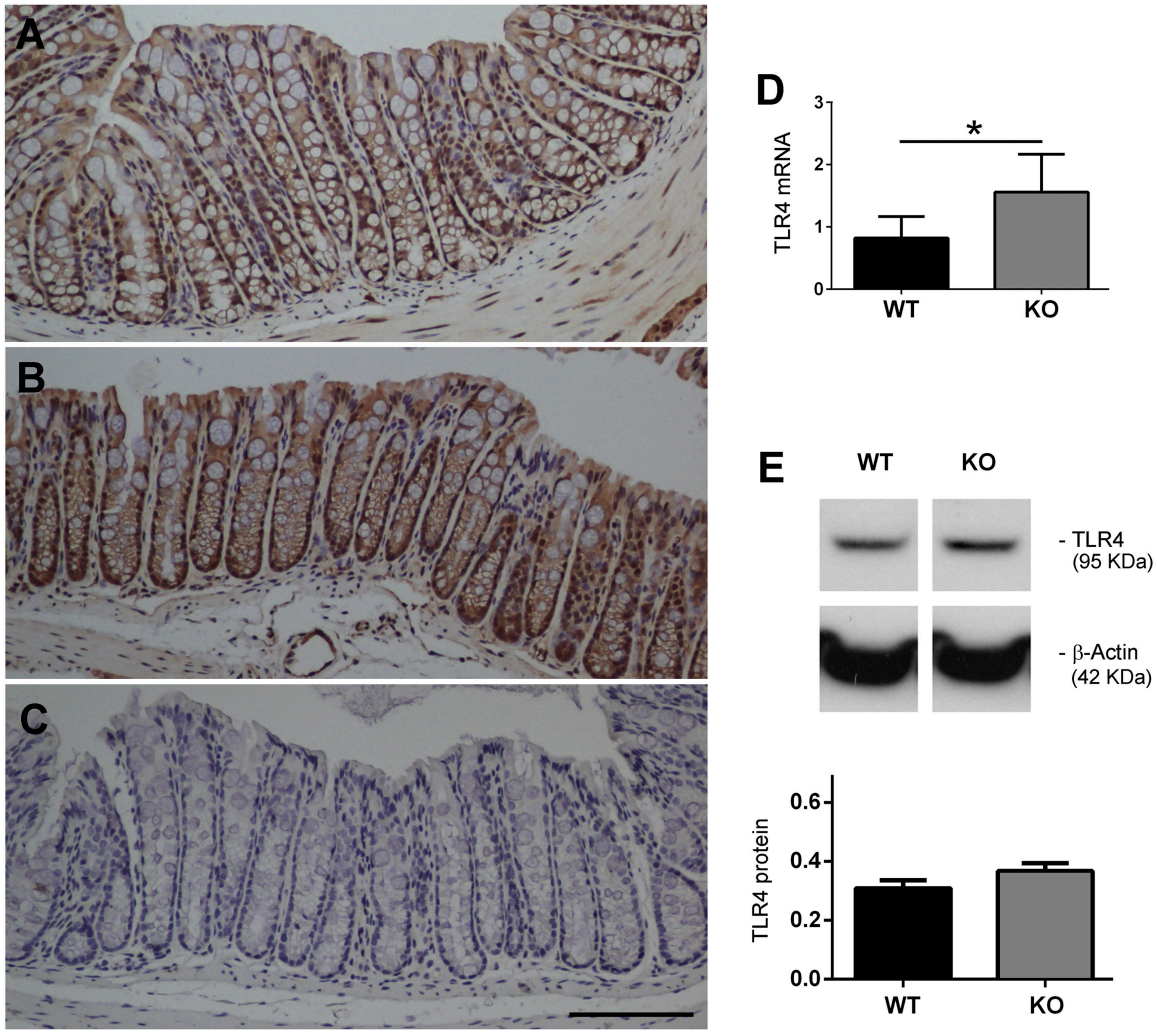
**Expression of TLR4 is elevated in AM KO mice**. TLR4 expression was assessed by immunohistochemistry **(A–C)**, qRT-PCR **(D)**, and Western blotting **(E)**. Colon sections from WT **(A)** and KO **(B)** mice were stained with antibodies against TLR4. Staining was more intense in KO mice. Absence of the primary antibody was used as a negative control **(C)**. Bar = 100 μm. TLR4 mRNA expression was assessed by qRT-PCR **(D)**. Bars represent the mean TLR4/18S quotient ± SEM. Kruskal-Wallis test; ^*^P < 0.05. TLR4 protein was measured by Western blotting, using β-Actin as a loading control **(E)**. Bars represent the normalized TLR4/ β-Actin value ± SEM. 20-week old WT (*n* = 8) and KO (*n* = 8) mice have been included.

Colon tissue sections from WT and KO mice were immunostained with an antibody against TLR4. In the WT mice the immunoreactivity was mainly found, as expected, in the epithelial cells of the colonic mucosa (Figure 3A). In the KO mice the distribution was the same but staining intensity was higher than in their WT counterparts (Figure 3B). Adjacent sections stained in the absence of primary antibody were used as a negative control (Figure 3C). We also measured colonic TLR4 mRNA expression levels (Figure 3D) in 20-week-old mice (*n* = 8 WT, *n* = 8 KO) and these were significantly augmented in KO mice when compared to WTs (*P* < 0.05). Protein levels were also measured by Western blotting (Figure 3E). Although there was some apparent increase of TLR4 immunoreactivity in KO mice, the differences did not reach statistical significance.

### Lack of adrenomedullin worsens colitis symptoms in AOM- and DSS-treated mice

One of the initial aims of this work was to analyze the impact of the lack of AM and PAMP on colitis-associated CRC initiation and progression using a research paradigm consisting in the injection of AOM and 3 weekly cycles of DSS. However, our KO mice experienced very extreme symptoms and the protocol had to be finished at this point. But the experience allowed us to investigate the effect of *adm* abrogation on the severity of DSS-induced colitis.

**Figure 4:**
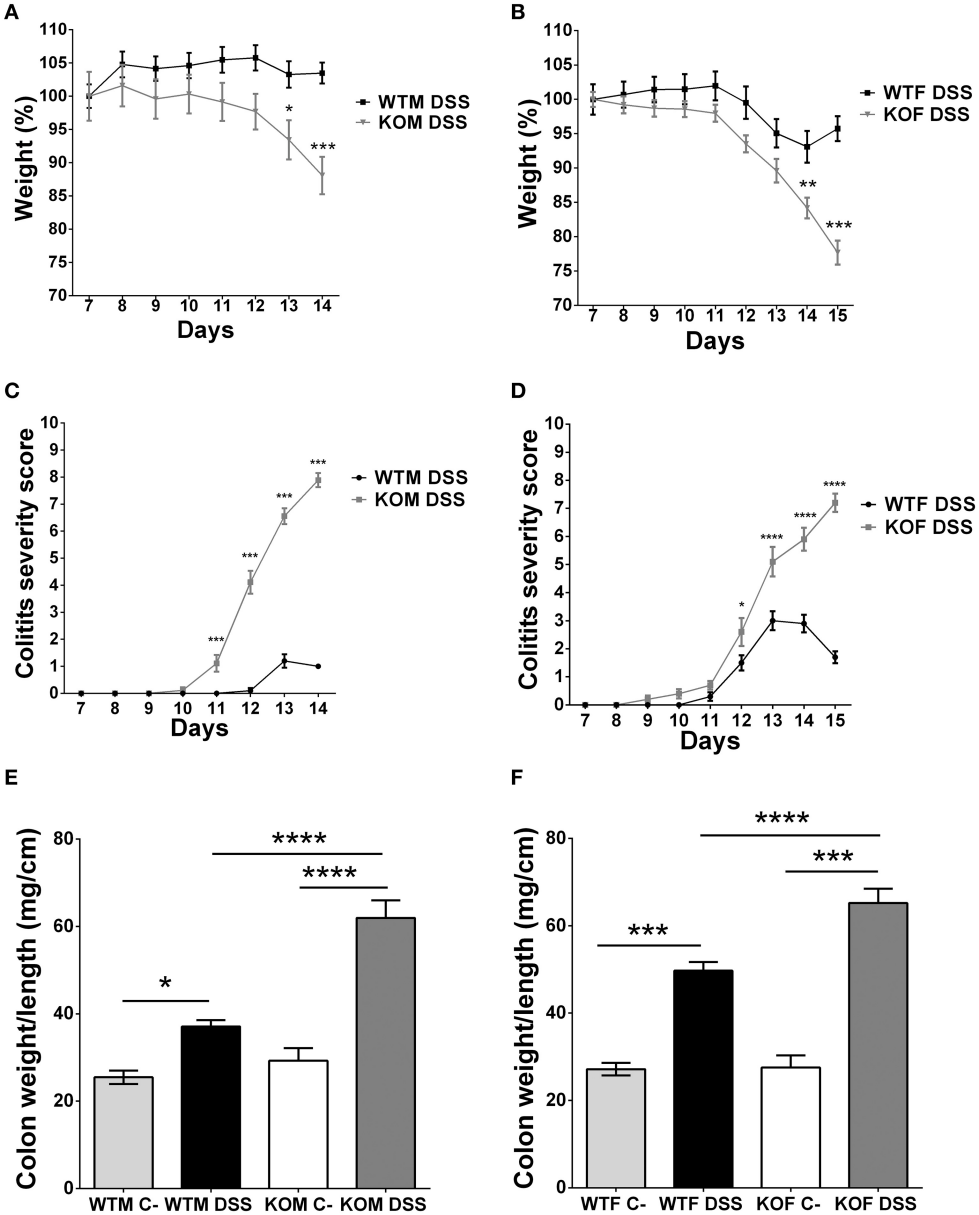
**Clinical assessment of colitis and macroscopic evidence of inflammation in DSS-treated mice**. Body weight changes were recorded daily in males **(A)** and females **(B)**. Lack of AM results in a significant weight loss. KOF experience a higher percentage of weight loss. Colitis symptoms were scored on a 0-12 point scale in males **(C)** and females **(D)**. KO animals exhibited severe colitis symptoms reaching higher scores than their WT counterparts. DSS treatment caused local inflammation of the colon in both males **(E)** and females **(F)**. There is a significant increase in the weight/length ratio of the colon in all DSS-treated mice when compared with their respective controls. However, this inflammatory response is more pronounced in *adm* deficient mice. Data are shown as mean ± SEM. ANOVA + Tukey's MCT; ^*^P < 0.05; ^**^P < 0.01; ^***^P < 0.001; ^****^P < 0.0001. Same abbreviations as in Figure 1.

KO mice treated with DSS experienced a profound and sustained weight loss (more than 30% in some cases) (*P* < 0.001) (Figures 4A,B), and extreme colitis symptoms such as dehydration, diarrhea, prolapses, and rectal bleeding compared to their WT counterparts (Figures 4C,D) (*P* < 0.001). In contrast, WTM maintained body weight (Figure 4A) and did not suffer major clinical symptoms (Figure 4C). On the other hand, WTF experienced light weight loss (less than 10%) (Figure 4B) and some colitis symptoms (Figure 4D). The colon weight/length ratio was recorded in both males (Figure 4E) and females (Figure 4F). There were no significant differences between the genotypes in untreated animals while treatment with DSS always resulted in a significant increase of this ratio. The increase was much higher in KO mice than in their WT littermates (*p* < 0.0001).

### Lack of adrenomedullin increases histopathological score and the loss of histological colonic structure in DSS treated mice

**Figure 5:**
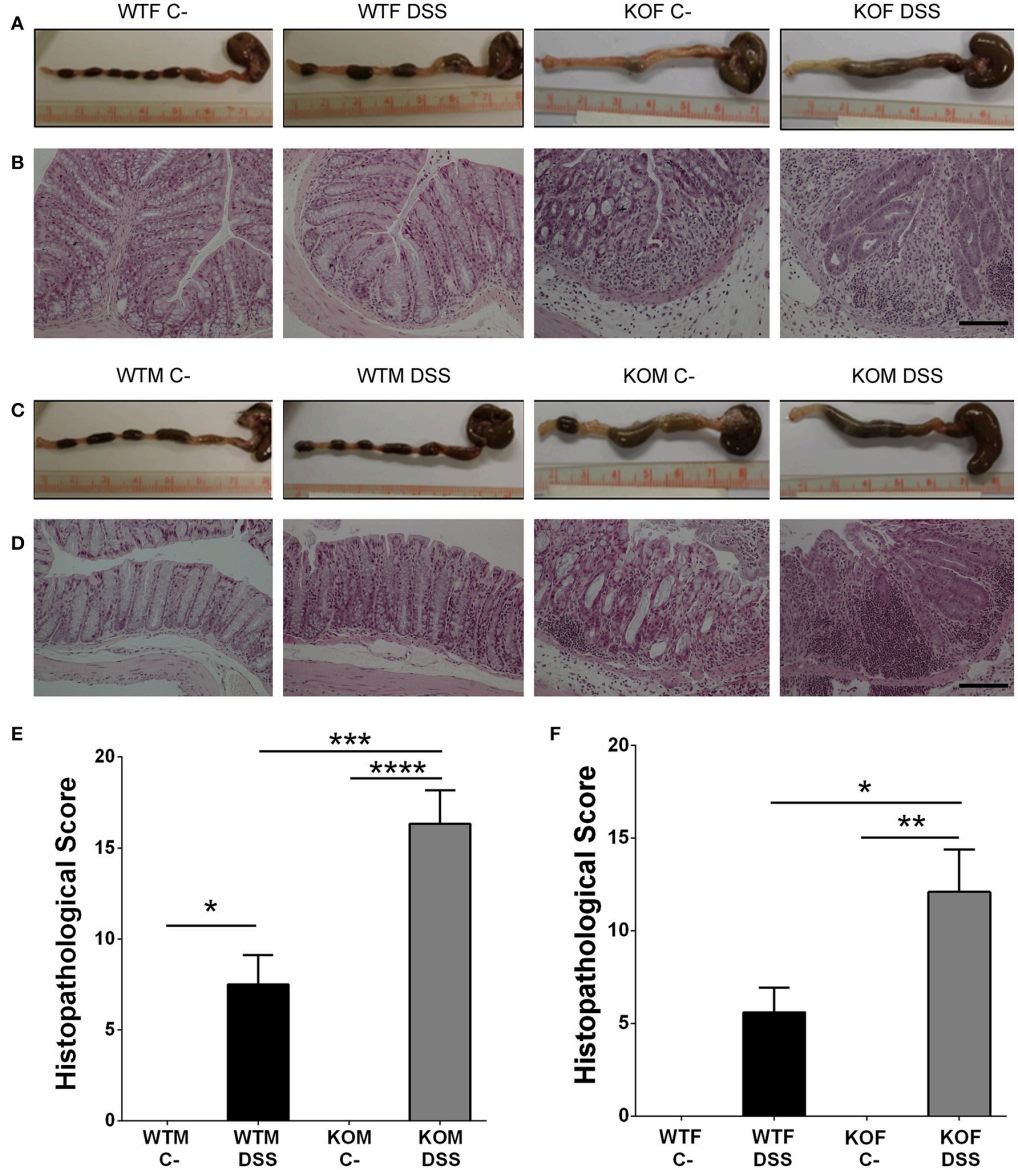
**Effect of endogenous AM on colon acute injury by DSS**. Representative macroscopic **(A,C)** and histological **(B,D)** appearance of the colon in females **(A,B)** and males **(C,D)** of the four experimental groups: untreated WTF (WTF C-), DSS-treated WTF (WTF DSS), untreated KOF (KOF C-), and DSS-treated KOF (KOF DSS). In untreated animals the characteristic oval shape and size of mice feces can be appreciated. However, DSS administration caused the presence of diarrhea, with bloody feces. Histological analysis of the colon of mice treated with DSS confirmed the macroscopic study data showing large areas of lymphocyte infiltrates in the mucosa and submucosa, being the damage greater in DSS-treated KOs with loss of tissue architecture, inflammation, and a more prominent infiltration when compared with WTs. Untreated KOs did not show any abnormality in the microscopic structure of the colon. Bars = 100 μm. The histopathological score was calculated in both males **(E)** and females **(F)**. All animals treated with DSS presented a severe inflammation in the colon, which was more severe among the KO mice. Data are shown as mean ± SEM. ANOVA + Tukey's MCT; ^*^P < 0.05; ^**^P < 0.01; ^***^P < 0.001; ^****^P < 0.0001. Same abbreviations as in Figure 1.

The macroscopic inspection of colon and rectum provided evidence of different degrees of colonic inflammation in DSS-treated animals. Treated KO mice presented more hemorrhagic areas (Figures 5A,C) than their WT littermates. Histological examination of the colonic sections of DSS treated mice revealed the presence of inflammatory cells restricted to isolated areas in WT animals, while most of the tissue maintained its micro architecture (Figures 5B,D). In KO animals a transmural inflammation involving all layers of the colon wall was observed, with massive infiltration in the mucosa and submucosa, and destruction of the crypts (Figures 5B,D). Histopathological scores were calculated and they revealed that DSS administration caused a significant increase in the pathological characteristics of both genotypes (Figures 5E,F), being this pathology much more severe in KO than in WT animals (*P* < 0.001 for males, *p* < 0.05 for females).

### Endogenous adrenomedullin reduces the expression of inflammatory cytokines in DSS colitis

**Figure 6:**
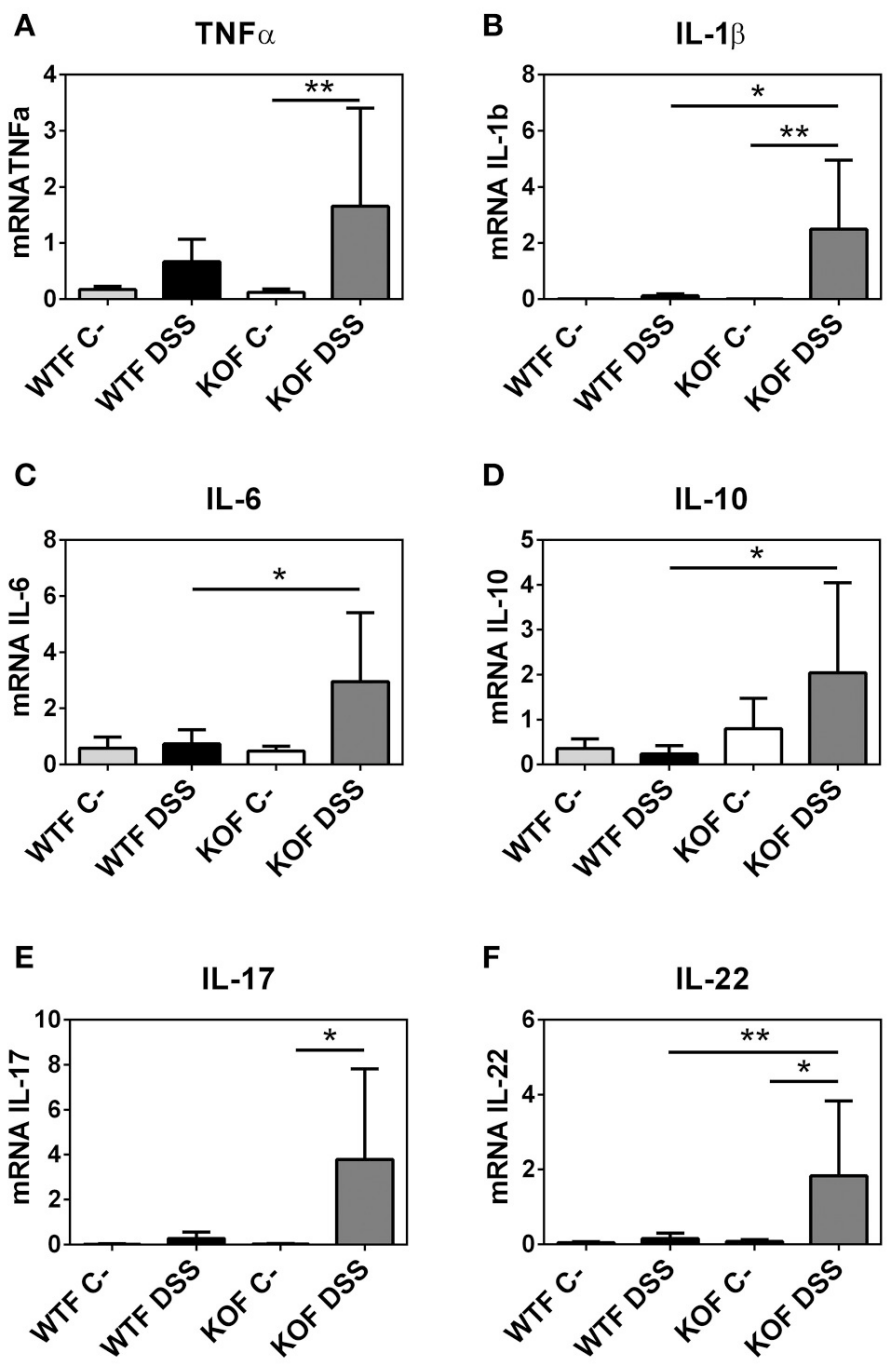
**Effects of endogenous adrenomedullin (AM) on the mucosal inflammatory responses during acute episode of colitis caused by DSS administration in females**. Expression of the principal pro- and anti-inflammatory cytokines **(A–F)** were evaluated by qRT-PCR in colon biopsies. Lack of AM resulted in a significant increase in the Th1 and Th2 cytokines when compared with their respective control mice and with their WT littermates. Surprisingly, DSS treatment did not cause any significant variation among the levels of expression of these cytokines in the DSS-treated WTs. All data were normalized by their 18S value. Data are shown as mean ± SEM. ANOVA + Tukey's MCT; ^*^P < 0.05; ^**^P < 0.01. Same abbreviations as in Figure 4.

To define the potential of endogenous AM to function as an anti-inflammatory agent we evaluated the mRNA expression levels of different cytokines (Figure 6 and Figure 3 of Supplementary Material).

In KOM, there was an increase in TNF-α (*P* < 0.0001), IL-1β (*P* < 0.001), and IL-6 (*P* < 0.01) levels after DSS administration compared with their sham group. In contrast, levels of these cytokines did not experience any variation in DSS treated WTM when compared with their untreated littermates, and were significantly lower when compared with KOM (*P* < 0.001 for TNF-α, *P* < 0.05 for IL-1β, *P* < 0.01 for IL-6). Moreover, levels of the anti-inflammatory cytokines IL-17 and IL-22 were also significantly augmented in treated KOM when compared with DSS treated WTM (*P* < 0.05 for IL-17, *P* < 0.05 for IL-22) and when compared with their sham littermates in the case of IL-22 (*P* < 0.05). Levels of IL-10 did not experience any significant variation in males (Figure 3 of Supplementary Material).

Among females, few variations were observed between DSS treated and untreated WT animals. DSS increased pro- and anti-inflammatory cytokine expression in KOFs when compared with their untreated controls (*P* < 0.01 for TNF-α, *P* < 0.01 for IL-1β, *P* < 0.05 for IL-17, and *P* < 0.05 for IL-22) and with their WT counterparts (*P* < 0.05 for IL-1β, *P* < 0.05 for IL-6, *P* < 0.05 for IL-10, and *P* < 0.01 for IL-22) (Figure 6).

### Lack of adrenomedullin does not modify the expression of adhesion molecules in the colon

In an attempt to identify the impact of AM in epithelial integrity we examined gene expression levels of different adhesion molecules (Figures 4, 5 of Supplementary Material). No significant change was observed in the gene expression patterns of these molecules with the exception of a significant decrease of β-catenin caused by DSS administration in KOF (*P* < 0.05) (Figure 5 of Supplementary Material).

## Discussion

In this study we have shown that elimination of the gene coding for AM in mice results in gut microbiota changes. The initial goal of linking lack of AM with colorectal cancer initiation and progression could not be reached with this methodology because AM deficiency results in an unexpected overreaction to DSS-induced colitis and the experimental protocol had to be terminated after the first DSS cycle. A possible alternative to study the role of this peptide in CRC initiation and progression is to use a less aggressive model, as those based on diets which recapitulate levels and length of exposure to nutrients linked to relative risk for human sporadic colon cancer (Yang et al., 2008). Nevertheless, this study provides new data on the influence of AM on colitis symptoms and colitis-related inflammation.

Previous studies have shown a strong positive effect using AM as treatment for colitis symptoms in rodents (Ashizuka et al., 2009) and humans (Ashizuka et al., 2013). However, none of the previous studies was able to investigate the opposite situation; i.e., the impact of the lack of endogenous AM on colitis. The scientific interest for this investigation is 2-fold. First, it provides a confirmation of AM's beneficial role in intestinal pathologies. And second, a particular single nucleotide polymorphism (SNP) close to the *adm* gene was found to be responsible for a reduction in the circulating levels of AM (Cheung et al., 2011) and to correlate with cancer susceptibility (Martínez-Herrero and Martínez, 2013). Therefore, it would be interesting to test whether carriers of this SNP are more susceptible to develop chronic inflammatory intestinal disorders or have worse symptoms.

This study shows that lack of endogenous AM correlates with an exacerbation of colitis symptoms at all levels suggesting that AM prevented the symptoms of the disease in line with previous experiments by the group in Sevilla (Talero et al., 2008). Microscopic analysis of colonic sections confirmed data from macroscopic observations reflecting an aggravation of mucosal disruption, edema, and inflammatory infiltration in KO mice. These results are in line with previous papers which demonstrated that AM exerts a protective action in DSS-induced experimental IBD (Ashizuka et al., 2009).

Previous studies have described that the DSS-induced colitis model induced high amounts of Th1 cytokines (TNF-α, IL-1β, IL-6) (Ashizuka et al., 2009). These cytokines exert potent proinflammatory effects that, when uncontrolled, can lead to tissue injury. Our cytokine determination demonstrated that DSS administration induces an inflammatory response in the colon of KO animal. Surprisingly, no elevation was observed in DSS-treated WT mice when compared with their respective controls. But in DSS-treated KO mice the response was very strong, suggesting that endogenous AM collaborates to maintain basal levels of these cytokines and to prevent an inflammatory overreaction. Previous research in AM supports our findings of the role of this peptide in immunoregulation (Wong et al., 2005; Carrizo et al., 2007).

In contrast with previous results, which have shown that DSS produces changes in the expression of tight and adherence junctions (Poritz et al., 2007; Ashizuka et al., 2009; Mennigen et al., 2009), we did not find any variation on the levels of these molecules. It is difficult to understand the reason for this discrepancy. The only difference is that we injected our mice with AOM before treating them with DSS. Future experiments would address this issue in more depth.

Another explanation for the exacerbation of colitis symptoms observed in KO animals is the alteration in gut microbiota that these mice suffer under physiological conditions. To our knowledge, this is the first study were the effects of AM deficiency on gut microbiota composition has been tested. Our results point to an important effect of this antimicrobial peptide in the control of gut bacteria composition which could offer a mechanistic insight into the pathogenesis of DSS-induced colitis and also could suggest several microbial interventions to be used in preventing or managing this condition.

The lack of AM did not translate into significant changes at higher taxonomic levels, suggesting that the worsening of DSS-induced colitis and inflammation observed in KO mice is more related to changes in lower taxonomic levels and diversity, as occurs in other metabolic pathologies such as obesity (Villanueva-Millán et al., 2015). It is also interesting to point out that some of the results obtained differ depending on sex. In this line, IBD seems to be more prevalent in women (Burisch et al., 2013) and it has been hypothesized that this situation might be related with female sex hormones since oral contraceptives are considered a risk factor for IBD (Ponder and Long, 2013). These data seem to indicate a potential link between sex-specific regulation of microbiota and IBD.

A previous study demonstrated that AM had antimicrobial activity against gut *B. fragilis, E. coli* and *H. pylori* (Allaker et al., 1999). As expected, we did not detect the presence of these species either in WT or KO mice, as these animals were maintained under SPF barrier conditions. However, we observed that the lack of AM was associated with a decreased abundance of two beneficial bacteria such as *L. gasseri* and *B. choerinum* (and also of the *Bifidobacterium* genus), which could suggest that the decrease in the numbers of these bacteria is associated with the worsening of DSS-induced colitis observed in KO mice. In fact, *L. gasseri* has anti-inflammatory activity that reduces the severity of colitis in a mouse model (Carroll et al., 2007). Also, administration of certain strains of *Lactobacillus* and *Bifidobacterium* improved DSS-induced colitis (Toumi et al., 2014).

In contrast, we observed a significant increase in the abundance of two bacteria belonging to the *Firmicutes* phyla (*Clostridium scindens* and *Christensenella minuta*) as well as in *B. vulgatus* levels, a weak inducer of intestinal inflammation that belongs to the *Bacteroidetes* phylum. The actions of *Bacteroides* in the pathogenesis of IBD has received attention (Liang et al., 2014). However, the role of *B. vulgatus* in these pathologies is controversial. Thus, while some studies demonstrated that its abundance was rather decreased among IBD patients (Takaishi et al., 2008) and that it was able to prevent the development of *Escherichia coli*-induced colitis (Sydora et al., 2007), others have reported that the administration of *Bacteroides*, and especially the administration of *B. vulgatus* aggravates colitis (Kishi et al., 2000). In our study, the increase observed in KO mice suggests a role of this bacterium in intestinal inflammation and also in the worsening of colitis induced by the lack of AM.

Surprisingly, a significant increase in the abundance of *Faecalibacterium prausnitzii*, an important member of mucosa-associated *Firmicutes*, was observed in male KO mice. Previous studies had reported that a reduction of *F. prausnitzii* was associated with the recurrence of ileal Crohn's disease (Sokol et al., 2008) and oral administration of this species can reduce the severity of experimental colitis in mice. As this phenomenon was not observed in female mice, the increase observed in males could suggest a compensatory mechanism to ameliorate the damage induced by AM deficiency. In fact, inflammation and severity was higher in females, where no increases in *F. prausnitzii* were observed.

Therefore, we can conclude that AM deficiency is clearly associated with changes in microbiota composition. This microbiota changes may predispose mice to suffer intestinal inflammation and worse DSS-induced colitis symptoms. But variations in microbiota may have another effect through changes in colonic TLR4 gene expression, although we have not able to show a clear increase of TLR4 protein by Western blotting. Hasegawa et al. (2010) demonstrated that immunostimulatory activity of the intestinal microflora varied during mouse development, evolving from a largely stimulation through TLR4 during breast-feeding to a TLR4-independent process after weaning. In AM deficient mice microflora there was a higher proportion of δ*-Proteobacteria*, which may be stimulating TLR4 in the colon and thus promoting its expression as it happened with mice during breast-feeding (Hasegawa et al., 2010). In addition, KO mice exhibited higher levels of other Gram negative bacteria that are able to stimulate TLR4 and increase its expression, such as *Clostridium* (Scarpa et al., 2011), *B. vulgates* (Haller et al., 2004), *Acinetobacter* (Erridge et al., 2007), or *Pseudomonadaceae* (Korneev et al., 2015). Today it is widely accepted that in normal intestinal epithelial cells, TLR4 is marginally expressed but under pathological conditions such as IBD and CRC an overexpression of this receptor occurs (Yesudhas et al., 2014). However, it has not been well established whether this TLR4 elevation occurs prior to IBD breakout or is a consequence of the disease. Our results seem to support the idea that an altered microbiota composition and a modified gene expression of TLR4 would precede and favor the appearance of the pathology.

To confirm the causal relationship between AM deficiency, microbiota changes, and colitis induction, manipulation of the gut microbiota should be performed, as described in other animal models (Li et al., 2016; Tian et al., 2016).

In conclusion, using our KO animals we have demonstrated that lack of endogenous AM causes an evident dysbiosis and an elevated TLR4 expression which might lead to a worse prognosis during colitis episodes.

## Author contributions

SM-H, IL, JN-I, MV-M, ER-F, PP-M, and JO: Performed experiments and interpreted data; SM-H and AM: Designed the study; AM: Wrote the manuscript and provided funding. All authors read and approved the final version of the manuscript.

## Funding

This study was supported by a grant from the Instituto de Salud Carlos III (PI13/02166), cofinanced by FEDER to AM; by a predoctoral fellowship from the Junta Provincial de La Rioja de la Asociación Española Contra el Cáncer (AECC) to SM-H; and by a predoctoral fellowship from Consejería de Industria, Innovación y Empleo (Government of La Rioja) to MV-M. Sponsors had no participation in study design, collection, analysis, and interpretation of the data.

### Conflict of interest statement

The authors declare that the research was conducted in the absence of any commercial or financial relationships that could be construed as a potential conflict of interest.
