# Supplementary material for: Lack of Adrenomedullin Results in Microbiota Changes and Aggravates Azoxymethane and Dextran Sulfate Sodium-Induced Colitis in Mice
Source: Front Physiol. 2016 Nov 30;7:595. doi: 10.3389/fphys.2016.00595 (PMC5127798; doi:10.3389/fphys.2016.00595)
Supplement: Supplementary file 1 [file DataSheet1.PDF]

Supplementary material:

Table 1. Primer sequences used for quantitative real time PCR measurements. The annealing temperature was 60ºC for all primers.

Gene	Primer	Sequence (5'⟶3')	Expected Amplicon Size
18S	Sense	ATG CTC TTA GCT GAG TGT CCC G	110 bp
	Antisense	ATT CCT AGC TGC GGT ATC CAG G	
Adrenomedullin	Sense	ATT GAA CAG TCG GGC GAG TA	130bp
	Antisense	CTT GGTCTT GGG TTC CTC TG	
TNF-α	Sense	GCA CCA CCA TCA AGG ACT CA	51 bp
	Antisense	TCG AGG CTC CAG TGA ATT CG	
IL-1β	Sense	ACA CTC CTT AGT CCT CGG CCA	51 bp
	Antisense	TGG TTT CTT GTG ACC CTG AGC	
IL-6	Sense	ATG GAT GCT ACC AAA CTG GAT	139 bp
	Antisense	TGA AGG ACT CTG GCT TTG TCT	
IL-10	Sense	AGG CGC TGT CAT CGA TTT CT	62 bp
	Antisense	CTC TTC ACC TGC TCC ACT GC	
IL-17	Sense	TCC AGA AGG CCC TCA GAC TA	239 bp
	Antisense	AGC ATC TTC TCG ACC CTG AA	
IL-22	Sense	TGC GAT CTC TGA TGG CTG TC	256 bp
	Antisense	CCT CGG AAC AGT TTC TCC CC	
ZO-1	Sense	GGA GCA GGC TTT GGA GGA G	163 bp
	Antisense	TGG GAC AAA AGT CCG GGA AG	
Occludin	Sense	GTC CTC CTG GCT CAG TTG AA	165 bp
	Antisense	CGG ACA TGG CTG ATG TCA CT	

JAM-A	Sense	AAC CCA TGG CTG ATT CCC AG	201 bp
	Antisense	TAG AGG ACG ACT TGG GGA GG	
B-catenin	Sense	TGT CCT GTG AAG CCC GC	225 bp
	Antisense	GCT TTT CTG TCC GGC TCC AT	
E-cadherin	Sense	GGA CAG CAA CAT CAG CGA AC	190 bp
	Antisense	GCT ACC ATC AAG AGC AGG CA	
Desmoglein-2	Sense	CCT CTT GCC ATT GAC CGA CT	56 bp
	Antisense	AAT GGC TGG GGT TCT GTG AG	
Connexin 26	Sense	GCT TAG TCG CTT AGT CGG CA	120 bp
	Antisense	CAC GGA GGC TTC TGG AGT TT	
B-actin	Sense	CCT AAG AGG AGG ATG GTC GC	230 bp
	Antisense	CTC AAC ACC TCA ACC CCC TC	
TLR4	Sense	ATG GCA TGG CTT ACA CCA CC	129 bp
	Antisense	GAG GCC AAT TTT GTC TCC ACA	

Figure 1. Metagenomic analysis of community composition in the four groups of experimental animals. Taxonomic levels of bacterial orders are shown. Data are presented as mean ± SEM. Wilcoxon test ; *: p<0.05, **: p<0.01 *vs* WTM; #: p<0.05 *vs* WTF. WT: Wildtype mice; KO: Knockout mice; WTM: male wildtype mice; WTF: female wildtype mice; KOM: male knockout mice; KOF: female knockout mice.

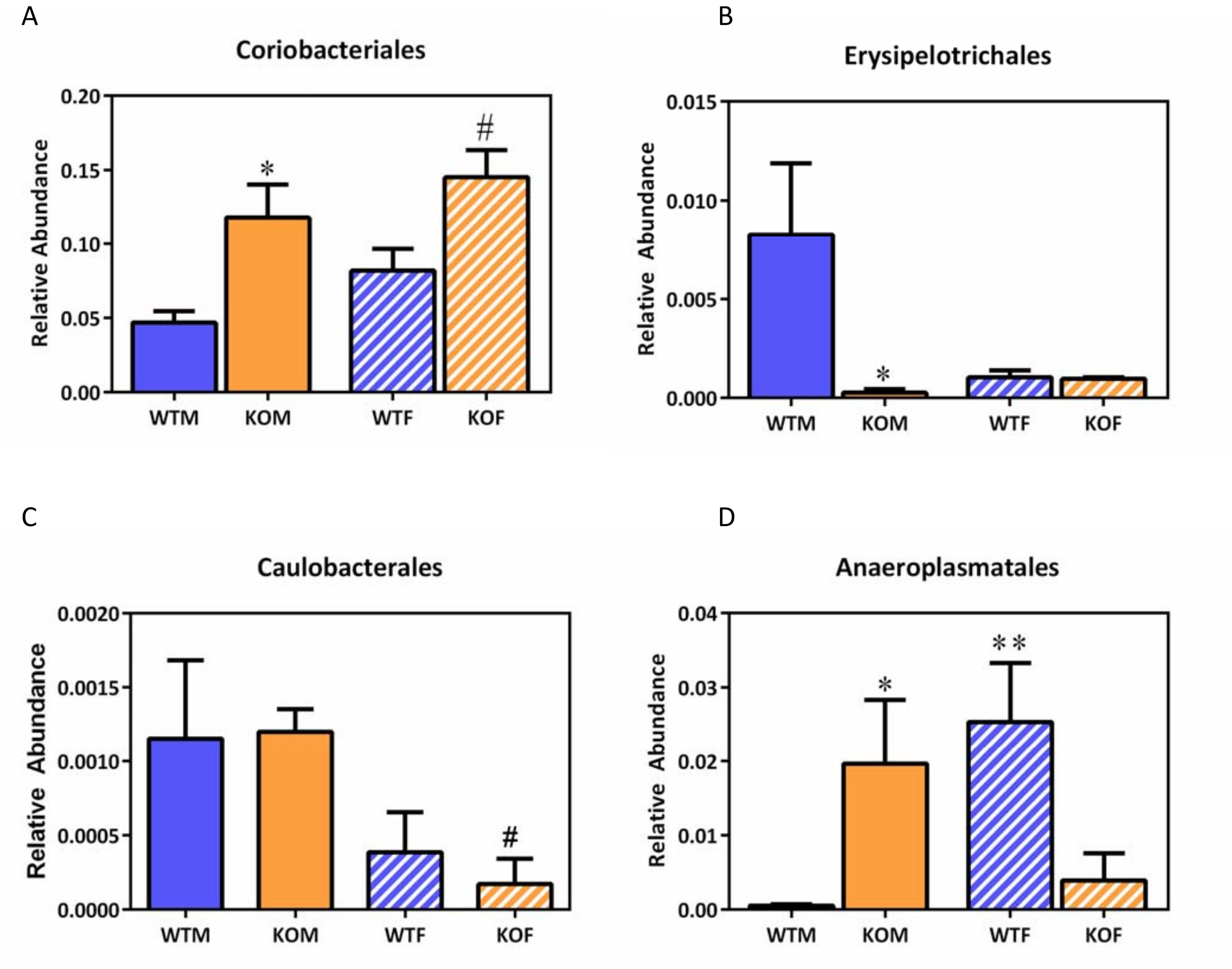

Figure 2. Relative abundance of *Lachnospiraceae bacterium* (A) and *Faecalibacterium prausnitzii* (B) in the four groups of experimental animals. Data are presented as mean ± SEM. Wilcoxon test; *: P<0.05 vs WTM. WT: Wildtype mice; KO: Knockout mice; WTM: male wildtype mice; WTF: female wildtype mice; KOM: male knockout mice; KOF: female knockout mice.

A

B

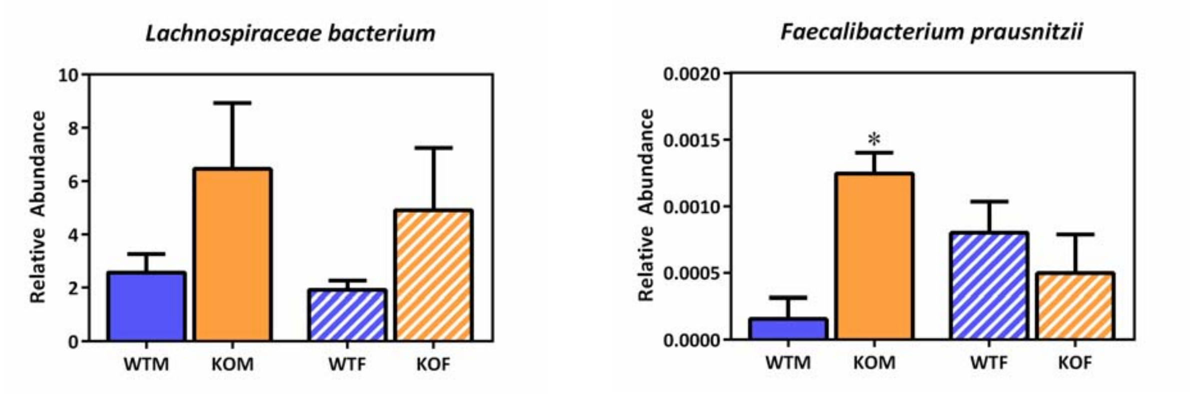

Figure 3. Effects of endogenous adrenomedullin (AM) on the mucosal inflammatory responses during acute episode of colitis caused by DSS administration in males. Expression of the principal pro- and anti-inflammatory cytokines were evaluated by qRT-PCR in colon biopsies. Lack of AM resulted in a significant increase in the Th1 and Th2 cytokines studied when compared with their respective control mice and with their WT littermates. Surprisingly, DSS treatment did not cause any significant variation among the levels of expression of these cytokines in the DSS-treated WTs. Data are shown as mean ± SEM. Kruskal-Wallis test; *: $P<0.05$. **: $P<0.01$. ***: $P<0.001$. ****: $P<0.0001$. WT: Wildtype mice; KO: Knockout mice; WTM C-: untreated male wildtype mice; WTM DSS: DSS-treated male wildtype mice; KOM C-: untreated male knockout mice; KOM DSS: DSS-treated male knockout mice.

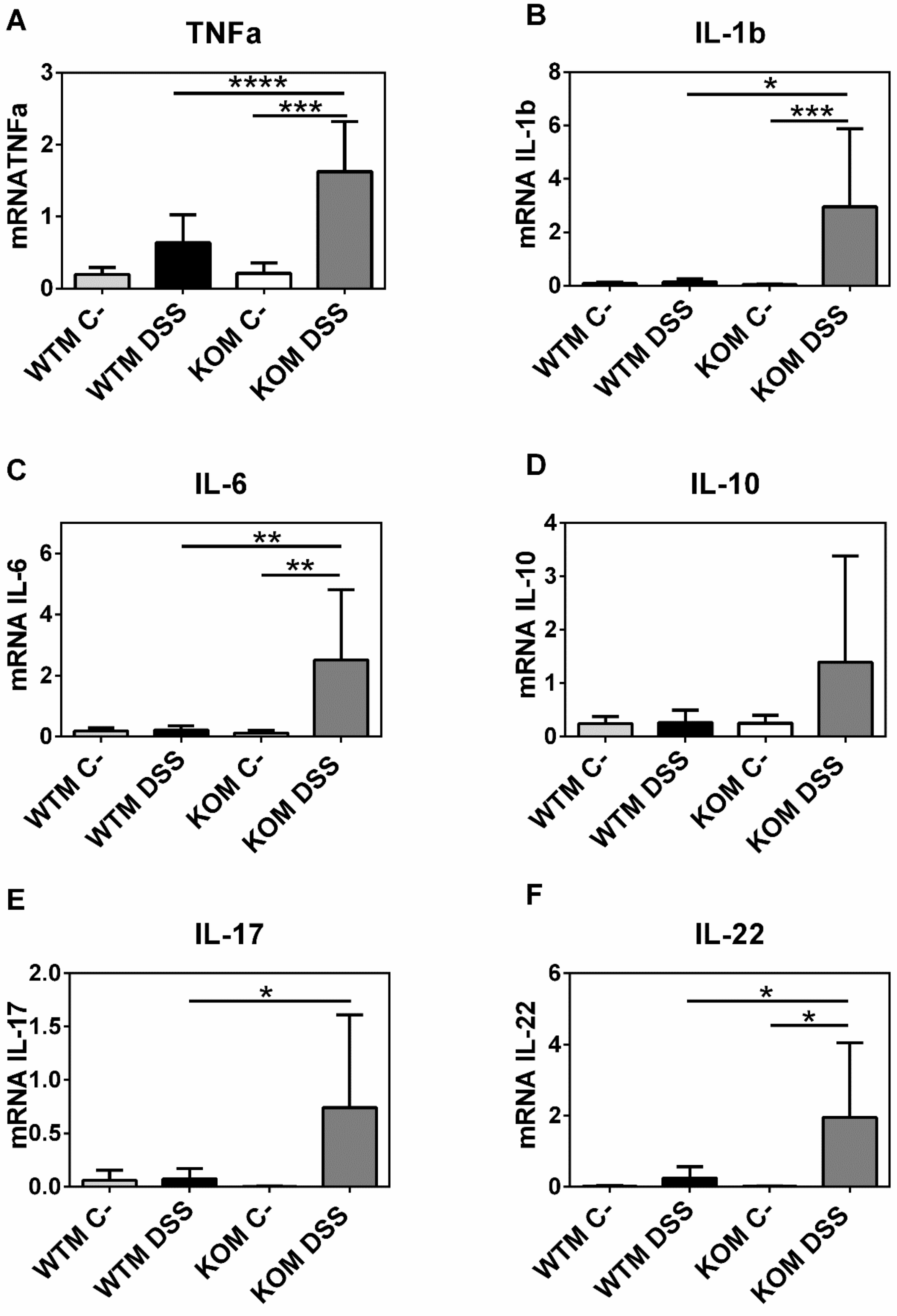
A
TNFa
mRNATNFa
0
1
2
3
****
***
WTM C-
WTM DSS
KOM C-
KOM DSS
B
IL-1b
mRNA IL-1b
0
2
4
6
8
*
***
WTM C-
WTM DSS
KOM C-
KOM DSS
C
IL-6
mRNA IL-6
0
2
4
6
**
**
WTM C-
WTM DSS
KOM C-
KOM DSS
D
IL-10
mRNA IL-10
0
1
2
3
4
WTM C-
WTM DSS
KOM C-
KOM DSS
E
IL-17
mRNA IL-17
0.0
0.5
1.0
1.5
2.0
*
WTM C-
WTM DSS
KOM C-
KOM DSS
F
IL-22
mRNA IL-22
0
2
4
6
*
*
WTM C-
WTM DSS
KOM C-
KOM DSS

Figure 4. Effects of endogenous adrenomedullin (AM) on the mucosal adhesion molecules in normal conditions and during acute episode of colitis caused by DSS administration in males. mRNA expression of the principal adhesion molecules was evaluated by qRT-PCR in biopsies of colon. No significant variation was observed among the studied groups. Data are shown as mean ± SEM. Kruskal-Wallis test. WT: Wildtype mice; KO: Knockout mice; WTM C-: untreated male wildtype mice; WTM DSS: DSS-treated male wildtype mice; KOM C-: untreated male knockout mice; KOM DSS: DSS-treated male knockout mice.

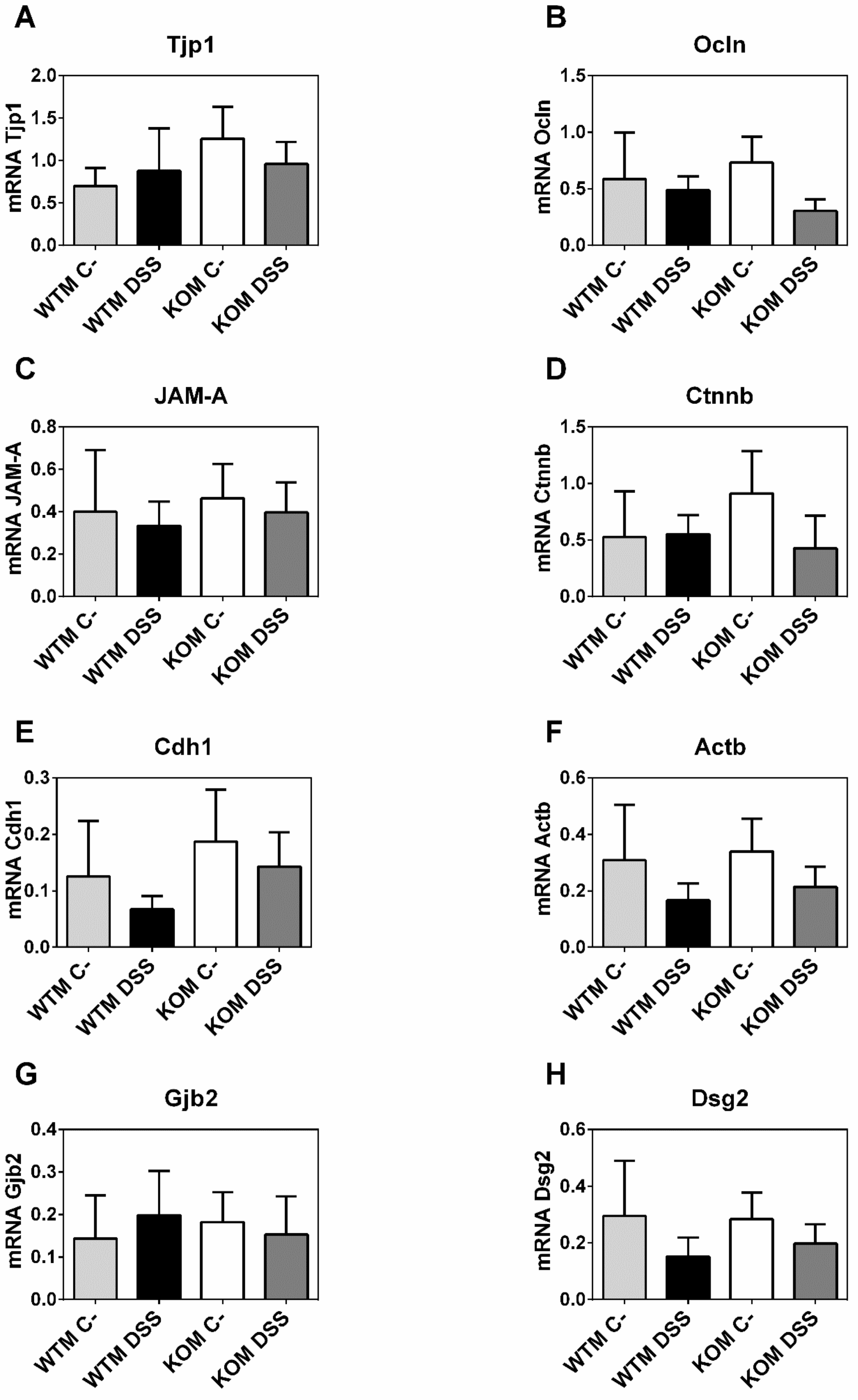

A
Tjp1
mRNA Tjp1
2.0
1.5
1.0
0.5
0.0
WTM C-
WTM DSS
KOM C-
KOM DSS
B
Ocln
mRNA Ocln
1.5
1.0
0.5
0.0
WTM C-
WTM DSS
KOM C-
KOM DSS
C
JAM-A
mRNA JAM-A
0.8
0.6
0.4
0.2
0.0
WTM C-
WTM DSS
KOM C-
KOM DSS
D
Ctnnb
mRNA Ctnnb
1.5
1.0
0.5
0.0
WTM C-
WTM DSS
KOM C-
KOM DSS
E
Cdh1
mRNA Cdh1
0.3
0.2
0.1
0.0
WTM C-
WTM DSS
KOM C-
KOM DSS
F
Actb
mRNA Actb
0.6
0.4
0.2
0.0
WTM C-
WTM DSS
KOM C-
KOM DSS
G
Gjb2
mRNA Gjb2
0.4
0.3
0.2
0.1
0.0
WTM C-
WTM DSS
KOM C-
KOM DSS
H
Dsg2
mRNA Dsg2
0.6
0.4
0.2
0.0
WTM C-
WTM DSS
KOM C-
KOM DSS

Figure 5. Effects of endogenous adrenomedullin (AM) on the mucosal adhesion molecules in normal conditions and during acute episode of colitis caused by DSS administration in females. mRNA expression of the principal adhesion molecules was evaluated by qRT-PCR in biopsies of colon. No significant variation was observed among the studied groups with the exception of β-catenin (D).DSS-treated KOF presented significantly lower levels of β-catenin. Data are shown as mean ± SEM. Kruskal-Wallis test ; *: $P<0.05$. WT: Wildtype mice; KO: Knockout mice; WTF C-: untreated female wildtype mice; WTF DSS: DSS-treated female wildtype mice; KOF C-: untreated female knockout mice; KOF DSS: DSS-treated female knockout mice.

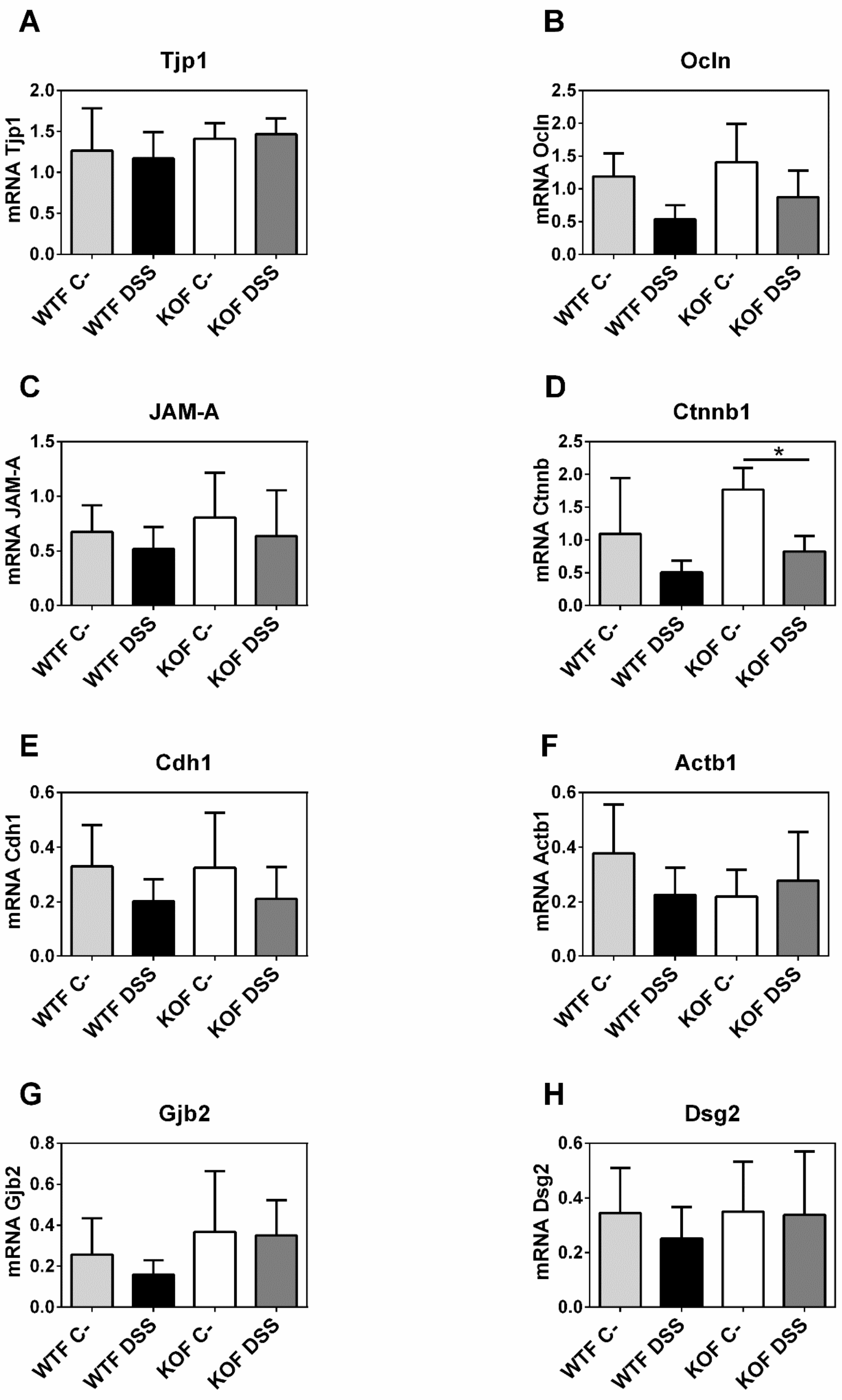
A
Tjp1
mRNA Tjp1
2.0
1.5
1.0
0.5
0.0
WTF C-
WTF DSS
KOF C-
KOF DSS
B
Ocln
mRNA Ocln
2.5
2.0
1.5
1.0
0.5
0.0
WTF C-
WTF DSS
KOF C-
KOF DSS
C
JAM-A
mRNA JAM-A
1.5
1.0
0.5
0.0
WTF C-
WTF DSS
KOF C-
KOF DSS
D
Ctnnb1
mRNA Ctnnb
2.5
2.0
1.5
1.0
0.5
0.0
*
WTF C-
WTF DSS
KOF C-
KOF DSS
E
Cdh1
mRNA Cdh1
0.6
0.4
0.2
0.0
WTF C-
WTF DSS
KOF C-
KOF DSS
F
Actb1
mRNA Actb1
0.6
0.4
0.2
0.0
WTF C-
WTF DSS
KOF C-
KOF DSS
G
Gjb2
mRNA Gjb2
0.8
0.6
0.4
0.2
0.0
WTF C-
WTF DSS
KOF C-
KOF DSS
H
Dsg2
mRNA Dsg2
0.6
0.4
0.2
0.0
WTF C-
WTF DSS
KOF C-
KOF DSS
